# A *CNTNAP1* Missense Variant Is Associated with Canine Laryngeal Paralysis and Polyneuropathy

**DOI:** 10.3390/genes11121426

**Published:** 2020-11-27

**Authors:** Anna Letko, Katie M. Minor, Steven G. Friedenberg, G. Diane Shelton, Jill Pesayco Salvador, Paul J. J. Mandigers, Peter A. J. Leegwater, Paige A. Winkler, Simon M. Petersen-Jones, Bryden J. Stanley, Kari J. Ekenstedt, Gary S. Johnson, Liz Hansen, Vidhya Jagannathan, James R. Mickelson, Cord Drögemüller

**Affiliations:** 1 Institute of Genetics, Vetsuisse Faculty, University of Bern, 3012 Bern, Switzerland; vidhya.jagannathan@vetsuisse.unibe.ch (V.J.); cord.droegemueller@vetsuisse.unibe.ch (C.D.); 2 Department of Veterinary and Biomedical Sciences, College of Veterinary Medicine, University of Minnesota, Saint Paul, MN 55108, USA; minork@umn.edu (K.M.M.); micke001@umn.edu (J.R.M.); 3 Department of Veterinary Clinical Sciences, College of Veterinary Medicine, University of Minnesota, Saint Paul, MN 55108, USA; fried255@umn.edu; 4 Department of Pathology, School of Medicine, University of California San Diego, La Jolla, CA 92093-0709, USA; gshelton@ucsd.edu (G.D.S.); jpesayco@ucsd.edu (J.P.S.); 5 Department of Clinical Sciences, Utrecht University, 3584 CM Utrecht, The Netherlands; p.j.j.mandigers@veterinair-neuroloog.nl (P.J.J.M.); P.A.J.Leegwater@uu.nl (P.A.J.L.); 6 Department of Small Animal Clinical Sciences, College of Veterinary Medicine, Michigan State University, East Lansing, MI 48824, USA; winkler.paige@gmail.com (P.A.W.); peter315@msu.edu (S.M.P.-J.); stanle32@msu.edu (B.J.S.); 7 Department of Basic Medical Sciences, College of Veterinary Medicine, Purdue University, West Lafayette, IN 47907, USA; kje0003@purdue.edu; 8 Department of Veterinary Pathobiology, University of Missouri, Columbia, MO 65211, USA; JohnsonGS@missouri.edu (G.S.J.); HansenL@missouri.edu (L.H.)

**Keywords:** *Canis familiaris*, whole-genome sequencing, rare disease, contactin, neurological disorder, Leonberger, Saint Bernard, Labrador retriever

## Abstract

Laryngeal paralysis associated with a generalized polyneuropathy (LPPN) most commonly exists in geriatric dogs from a variety of large and giant breeds. The purpose of this study was to discover the underlying genetic and molecular mechanisms in a younger-onset form of this neurodegenerative disease seen in two closely related giant dog breeds, the Leonberger and Saint Bernard. Neuropathology of an affected dog from each breed showed variable nerve fiber loss and scattered inappropriately thin myelinated fibers. Using across-breed genome-wide association, haplotype analysis, and whole-genome sequencing, we identified a missense variant in the *CNTNAP1* gene (c.2810G>A; p.Gly937Glu) in which homozygotes in both studied breeds are affected. *CNTNAP1* encodes a contactin-associated protein important for organization of myelinated axons. The herein described likely pathogenic *CNTNAP1* variant occurs in unrelated breeds at variable frequencies. Individual homozygous mutant LPPN-affected Labrador retrievers that were on average four years younger than dogs affected by geriatric onset laryngeal paralysis polyneuropathy could be explained by this variant. Pathologic changes in a Labrador retriever nerve biopsy from a homozygous mutant dog were similar to those of the Leonberger and Saint Bernard. The impact of this variant on health in English bulldogs and Irish terriers, two breeds with higher *CNTNAP1* variant allele frequencies, remains unclear. Pathogenic variants in *CNTNAP1* have previously been reported in human patients with lethal congenital contracture syndrome and hypomyelinating neuropathy, including vocal cord palsy and severe respiratory distress. This is the first report of contactin-associated LPPN in dogs characterized by a deleterious variant that most likely predates modern breed establishment.

## 1. Introduction

Laryngeal paralysis (LP) can result from trauma or neoplasia involving the recurrent laryngeal nerves, peripheral nerve disease, or a primary or secondary disease affecting the muscle or neuromuscular junction. Loss of normal function of the larynx leads to breathing difficulties, reduced exercise and heat tolerance, as well as an increased risk of aspiration pneumonia [1]. Laryngeal nerve disease results in degeneration and atrophy of intrinsic laryngeal muscles followed by decreased or absent movement of the attendant laryngeal cartilages. During breathing, these cartilages control airflow into and out of the trachea. Affected dogs have stridor, may have a change in vocalization, and difficulty breathing due to the flaccid laryngeal vocal folds and corniculate processes of the arytenoid obstructing the lumen of the airway [1]. Normal laryngeal function protects the airway by closing off the lumen to prevent aspiration of food or water. In LP-affected dogs, the vocal folds remain in a paramedian position, causing airway resistance and turbulence, instead of abducting, as they normally would, to open the airway during inspiration. Frequently, affected dogs suffering from LP are treated by crico- or thyro-arytenoid laryngoplasty surgery, to improve breathing and, therefore, quality of life [2]. As the recurrent laryngeal nerve axons are some of the longest in the body [3], LP is often reported as part of a more generalized length-dependent polyneuropathy (PN) complex, which manifests with additional signs including proprioceptive and motor abnormalities, slowly progressing pelvic limb weakness, and loss of limb muscle mass [4].

Various mostly breed-specific canine inherited neuropathies form a heterogeneous group of degenerative diseases affecting motor and/or sensory and autonomic peripheral nerves. This group includes mixed forms of LP and PN [5], i.e., the laryngeal paralysis and polyneuropathy complex (LPPN), which has variable ages of onset among and across several dog breeds (OMIA 001206-9615, OMIA 001292-9615). Late-onset forms, e.g., geriatric onset laryngeal paralysis polyneuropathy (GOLPP), are also observed in various breeds including Labrador retrievers [6]. Leonberger dogs are known to be susceptible to LPPN; recently, a short list of potentially pathogenic variants for neurological disorders in this breed derived from whole-genome sequencing has been presented [7]. To date, variants in *ARHGEF10* [8] and *GJA9* [9] have already been associated with certain forms of the disorder and designated with breed-specific names Leonberger polyneuropathy type 1 (LPN1; OMIA 001917-9615) and Leonberger polyneuropathy type 2 (LPN2; OMIA 002119-9615), respectively. These two variants, however, do not explain all the phenotypically described cases in Leonbergers [7]. The *ARHGEF10* variant has also been reported in the related Saint Bernard breed, but again it did not explain all LPPN cases [8]. Alaskan huskies, black Russian terriers, and Rottweilers with PN including LP and respiratory distress are known to have deleterious variants in the *RAB3GAP1* gene, a member of the RAB3 protein family implicated in regulated exocytosis of neurotransmitters and hormones (OMIA 001970-9615) [10,11,12]. Another major risk factor for canine LP recently described in miniature bull terriers and bull terriers is a variant in the *RAPGEF6* gene encoding a widely expressed nucleotide exchange factor whose function is not well understood (OMIA 002222-9615) [13].

In general, there are limits to precisely diagnosing neurological diseases in dogs in the clinic. For example, in a previous study [14], we noticed Leonbergers that were initially clinically diagnosed as polyneuropathy-affected, although, in fact, they were suffering from leukoencephalomyelopathy, a juvenile-onset neurodegenerative disorder of the CNS white matter with distinctive pathological features, caused by a recessive variant in the *NAPEPLD* gene (OMIA 001788-9615).

Our aim in this study was to identify additional causative genetic variants associated with younger-onset laryngeal paralysis and polyneuropathy (LPPN), by focusing on two closely related giant dog breeds [15], namely the Leonberger and Saint Bernard.

## 2. Materials and Methods

### 2.1. Ethics Statement

All animal experiments were performed according to local regulations, and all animals in this study were examined with the consent of their owners. The study was approved under IACUC protocol 1903-36865A at the University of Minnesota, the Michigan State University Institutional Animal Care and Use Committee (AUF number 01/11-009-00), and by the Cantonal Committee for Animal Experiments (Canton of Bern; permit 71/19) at the University of Bern.

### 2.2. Animal Selection

Data on 15,378 dogs from 243 breeds, 321 dogs of mixed or unknown heritage, and 62 wild canids were collected in three different sets for this study (Table 1 and Supplementary Table S1). The discovery cohort included 426 Leonbergers either showing signs of LPPN with an age of onset ≤5 years or healthy control dogs at ≥8 years of age, and 91 Saint Bernards either showing signs of LPPN with an age of onset ≤5 years or population control dogs with genome-wide association study (GWAS) data available from unrelated studies [15,16]. All 517 Leonbergers and Saint Bernards were genotyped for the *ARHGEF10* variant [8], and the Leonbergers were also genotyped for the *GJA9* variant [9], in order to include only dogs homozygous wild type for these loci. In addition, the Leonbergers were genotyped for a previously reported leukoencephalopathy-associated *NAPEPLD* variant [14], in order to rule out another known underlying neurological disease with similar clinical phenotype.

A validation cohort used for targeted genotyping of the newly discovered variant consisted of 1070 dogs with known LPPN phenotypes (Table 1 and Supplementary Table S1). There was no age of onset restriction for the cases in the validation cohort. Included in the validation cohort were 193 Labrador retrievers and seven mixed-breed dogs from an ongoing geriatric onset laryngeal paralysis polyneuropathy (GOLPP) study; these were used as an independent validation group (Table 1).

Finally, a population cohort consisting of 14,112 dogs, 58 wolves, two golden jackals, one Andean fox, and one dhole (Table 1 and Supplementary Table S1), with no available information about their health status, was used to determine the absence/presence and frequency of the described variant-associated haplotype across canids.

The information about age of onset of clinical signs in the LPPN-affected dogs was available for a subset of 770 dogs from the discovery and validation cohorts with detailed health information from three breeds (596 Leonbergers, 28 Saint Bernards, and 146 Labrador retrievers) The statistical significance of the differences between groups was evaluated with Student’s *t*-test and *p* < 0.05 was considered as significant.

### 2.3. Sample Preparation

Genomic DNA was isolated from EDTA blood samples, buccal swabs, or archived muscle biopsies by using either the Gentra PureGene kit (Qiagen, Hilden, Germany) or the Maxwell RSC Whole Blood DNA kit (Promega, Dübendorf, Switzerland).

Clinical cases of polyneuropathy and laryngeal paralysis were evaluated in three dogs homozygous for the studied *CNTNAP1* variant with available nerve biopsies; these included a 3-year-old Saint Bernard, a 3-year-old Leonberger, and a 9-year-old Labrador retriever (Supplementary Table S1). Three normal adult dog samples from Labrador retrievers were used as controls. The ages for the control dogs were 8–10 years of age. All the archived nerve specimens were obtained years prior to the identification of the *CNTNAP1* variant. Peroneal nerve specimens, pinned on cork discs to maintain length and orientation, were immersion-fixed in 2.5% glutaraldehyde in 0.1 M phosphate buffer before shipment. Upon receipt, the nerves were postfixed in 1% aqueous osmium tetroxide for 3 to 4 h before dehydration in a graded alcohol series and propylene oxide. After infiltration with a 1:1 mixture of propylene oxide and araldite resin for 4 h, nerves were placed in 100% araldite resin overnight before embedding in fresh araldite resin. Thick sections (1 µm) were cut with glass knives and either stained with toluidine blue prior to light microscopic examination, or stained with paraphenylenediamine prior to morphometry.

### 2.4. Axonal Size Frequency Distributions and G-Ratios

Axonal size-frequency distributions of myelinated fibers were performed on transverse sections of selected peroneal nerve biopsies determined to be adequately fixed, free from artifact, and with an intact perineurium. Images were obtained from a single section of each nerve biopsy using the Photoshop image analysis system. Profiles containing paranodal regions or Schmidt–Lanterman clefts were not included. Using a ×60 objective, the final magnification of the digitized image was equivalent to 1 pixel = 0.091 µm. Myelinated fibers were individually identified and selected prior to being sorted with an automated process into bins based on axonal area. G-ratios were calculated as the ratio between the diameter of the axon itself and the outer diameter of the myelinated fiber.

### 2.5. Single Nucleotide Polymorphism Array Genotyping and Imputation

The discovery cohort (426 Leonbergers and 91 Saint Bernards) was genotyped by using either the Axiom Canine Set A or HD arrays (Thermo Fisher Scientific, Waltham, MA, USA) or the Illumina CanineHD BeadChip array (Illumina, San Diego, CA, USA). Samples genotyped on lower single nucleotide polymorphism (SNP) density arrays were imputed with Beagle 4.1 [17,18], using a diverse reference dataset containing 526,045 variants in 49 wolves and 2871 dogs (including 65 Leonbergers and 23 Saint Bernards; Supplementary Table S1). The data were filtered to include only biallelic SNPs with a minor allele frequency ≥0.02, a per-SNP genotyping rate ≥95%, and a per-individual genotyping rate ≥95%. The reference dataset was phased on a per-chromosome basis, using Beagle 4.1 with default parameters of 10 iterations and an effective population size of 200. Next, a target dataset containing approximately 174,000 variants in 402 Leonbergers and 66 Saint Bernards (Supplementary Table S1) was filtered to include only biallelic SNPs with a minor allele frequency ≥0.02, and then checked for concordance with the filtered and phased reference dataset, using the Beagle 4 utility conform-gt [19]. Conforming sites of the target dataset were imputed to the reference dataset on a per-chromosome basis, using Beagle 4.1 with the following settings: window size 50 kb, overlap 3 kb, effective population size 200, and 10 iterations. The per-chromosome imputed data were concatenated and sorted, using VCFtools 0.1.13 [20]; variants with a Beagle 4.1 dosage R-squared (DR2) ≥0.7 were retained for downstream analysis. In total, we used imputed SNP data for 468 Leonberger and Saint Bernard dogs in this study.

To evaluate haplotypes across breeds, approximately 126,000 SNPs common across genotyping platforms were extracted from non-imputed SNP genotype data for 12,931 canids, which were either generated during this study or publicly available (Supplementary Table S1) and phased with Beagle 4.1 as described above.

### 2.6. Genome-Wide Association Study and Fine-Mapping

The discovery cohort from the two breeds combined contained 517 dogs (144 LPPN-cases and 373 controls). Quality control filtering steps of the imputed SNP array genotyping data were carried out by using PLINK v1.9 [21]. The dataset was pruned for low minor allele frequency (0.05) and failure to meet Hardy–Weinberg equilibrium (0.0001) and consisted of 289,553 markers. An across-breed genome-wide association study (GWAS) was performed with GEMMA v0.98 [22], using a linear mixed model including an estimated kinship matrix from centered genotypes to correct for the genomic inflation. The significance threshold was estimated by Bonferroni correction. Manhattan and Q–Q plots of the corrected *p*-values were generated in R environment v3.6.0 [23], using the qqman package [24]. Haplotypes around the significantly associated locus obtained from GWAS were constructed by using Beagle 4.1 for all canids with available SNP genotype data (*n* = 13,399) (Supplementary Table S1). All genome positions refer to the CanFam3.1 reference assembly.

### 2.7. Whole-Genome Sequencing

Whole-genome sequence (WGS) data of 716 publicly available dogs of 131 different breeds, and nine wolves [25] (Supplementary Table S2) were studied in order to identify the causative variant in the disease-associated region obtained by the GWAS. This set included 34 Leonbergers and two Saint Bernards diagnosed with a form of LPPN unexplained by the previously known variants in *ARHGEF10* [8], *GJA9* [9], *RAB3GAP1* [10,11,12] and *RAPGEF6* [13], as well as seven Leonbergers and one Saint Bernard used as controls. The sequence data analysis and calling of single nucleotide variants and small indels (SNVs), including the prediction of functional effects, were described previously [25]. The Integrative genomics viewer (IGV) software 2.8.2 [26] was used for visual inspection and screening for structural variants in the region of interest in the affected dogs’ WGS.

### 2.8. Targeted Genotyping

Polymerase chain reaction (PCR) and Sanger sequencing were used to validate and genotype the variant identified from WGS. PCR products from genomic DNA were amplified by using AmpliTaqGold360 MasterMix (Thermo Fisher Scientific), and the purified PCR amplicons were directly sequenced on an ABI3730 capillary sequencer (Thermo Fisher Scientific). The *CNTNAP1* missense variant (XM_548083.6:c.2810G>A) was genotyped, using the following primers: TCCCTTGCCCTCCCTATATC (forward) and AGTCCTAATGCCCTCTGCTG (reverse). The sequence data were analyzed by using Sequencher 5.1 software (GeneCodes, Ann Arbor, MI, USA).

### 2.9. Protein Predictions

The MutPred2 [27], PROVEAN [28] and PON-P2 [29] in silico prediction tools were used to predict biological consequences of the discovered variant on the encoded protein. All references to the canine *CNTNAP1* gene correspond to the accessions NC_006591.3 (NCBI accession), XM_548083.6 (mRNA), and XP_548083.3 (protein). The Genome Aggregation Database (gnomAD) [30] was searched for the corresponding variant in the human *CNTNAP1* gene (NP_003623.1).

### 2.10. Availability of Data and Material

The WGS are freely available at the European Nucleotide Archive (ENA). All accession numbers of the used genomes are available in the Supplementary Table S2. The sources of SNP array genotyping data published before are detailed in the Supplementary Table S1, and the dataset generated for this study is available from the corresponding author on reasonable request. All genome positions are reported with respect to the dog reference genome assembly CanFam3.1 and NCBI annotation release 105.

## 3. Results

### 3.1. Phenotype

The herein studied affected dogs showed generic signs of LPPN (Supplementary Table S1) with the key feature across breeds being breathing difficulty, often described as noisy or raspy breathing (Supplementary Video S1). Due to LP, 247 dogs (121 Leonbergers, 10 Saint Bernards, 114 Labrador retrievers, and 2 mixed-breed dogs) underwent an arytenoid lateralization surgery, 25 of which (ten Leonbergers, seven Saint Bernards, six Labrador retrievers, and two mixed-breed dogs) tested homozygous for the studied *CNTNAP1* variant (Supplementary Table S1). Additional clinical signs, which were noted variably among the dogs, included difficulty swallowing, changes in barking frequency and quality, high-stepping and uncoordinated gait, stumbling and tripping, exercise intolerance, and limb muscle atrophy.

### 3.2. Neuropathological and Morphometric Findings

**Figure 1:**
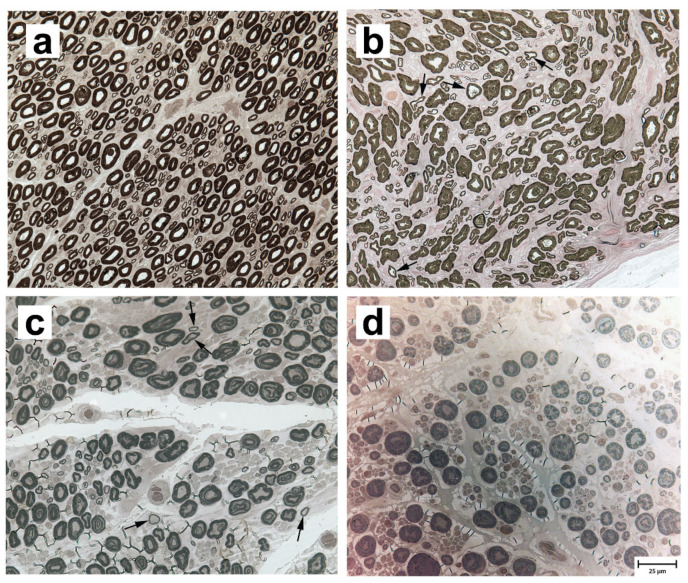
Paraphenylenediamine-stained resin sections from the peroneal nerves of four dogs. (**a**) An adult normal control Labrador retriever, (**b**) a nine-year-old LPPN-affected Labrador retriever, (**c**) a three-year-old LPPN-affected Leonberger, and (**d**) a three-year-old LPPN-affected Saint Bernard. All three LPPN-affected dogs were homozygous for the *CNTNAP1* variant. Arrows in (**b**) and (**c**) point to nerve fibers that are inappropriately thin for the axon diameters. Bar in lower right image indicates 25 µm and is valid for all images.

Peroneal nerve biopsies were evaluated from three archived normal adult Labrador retriever (8–10 years) samples (representative image in Figure 1a), and three LPPN-affected dog samples: a nine-year-old male Labrador retriever (Figure 1b), a three-year-old male Leonberger (Figure 1c), and a three-year-old male Saint Bernard (Figure 1d), all of which tested homozygous for the studied *CNTNAP1* variant. Compared to control nerve, pathological changes were similar among affected dogs of all three breeds and included a subjective decrease in the number of myelinated nerve fibers compared to control nerve (Figure 1b–d) with scattered inappropriately thin myelin sheaths for the axon diameter (best demonstrated in Figure 1b,c). The inappropriately thin myelinated fibers were not found in the nerves of control dogs. Myelin splitting and ballooning, onion-bulb formations, and axonal degeneration were not observed in any of the biopsies.

A histogram of axonal size-frequency distribution of the relative percentage of small (<5 µm) and large (>5 µm) myelinated nerve fibers is shown for the three control Labrador retrievers, the LPPN-affected Leonberger, and the LPPN-affected Labrador retriever (Supplementary Figure S1) described above. As only a partial nerve fascicle was available for the LPPN-affected Saint Bernard, those data were not included. The large and small nerve fibers are determined by the axon diameters and this does not refer to the thickness of the myelin sheath. Compared to the average values for the control Labrador retrievers, the affected Labrador retriever showed an increased population of small caliber nerve fibers and a decreased population of large caliber nerve fibers. In contrast, the affected Leonberger showed a decreased population of small fibers and an increased population of larger fibers. Calculated G-ratio, a quantitative measure of myelin thickness, was 0.586 ± 0.031 (range 0.552–0.609) for the control Labrador retrievers, 0.543 for the affected Leonberger, and 0.575 for the affected Labrador retriever.

### 3.3. Genome-Wide Association Study and Fine-Mapping

**Figure 2:**
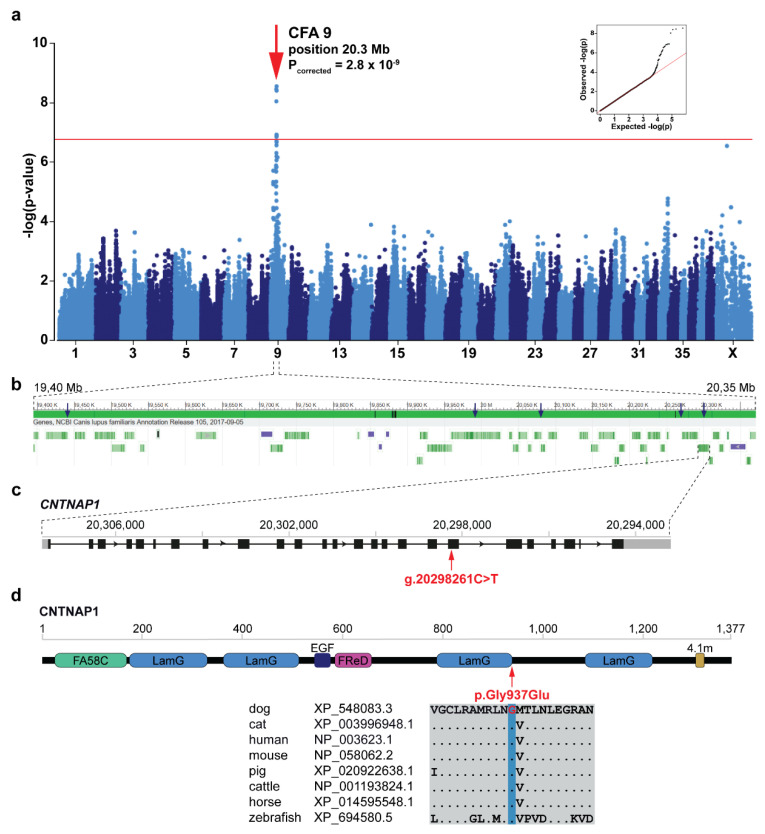
Identification of a new LPPN-associated locus and variant in Leonbergers and Saint Bernards. (**a**) Manhattan plot for the two-breed genome-wide association study (GWAS) using 144 LPPN-affected dogs and 373 normal control dogs indicates a signal with multiple associated single nucleotide polymorphisms (SNPs) on chromosome 9. The -log *p*-values for each SNP are plotted on the *y*-axis versus each canine chromosome on the *x*-axis. The red line represents the Bonferroni corrected significance threshold (−log(*p*-value) = 6.76). Inset: Corrected Q–Q plot confirms that the observed *p*-values of the best-associated markers have stronger association with the trait than expected by chance (null hypothesis, red line). (**b**) Gene content in the ~0.98 Mb region of interest. Blue arrows show the best-associated markers from GWAS. Green bars represent the different genes and violet bars represent lncRNAs. (**c**) Schematic representation of the *CNTNAP1* gene showing the variant (XM_548083.6:c.2810G>A) location in exon 18. (**d**) Schematic representation of the CNTNAP1 protein with its domains: coagulation factor 5/8 C-terminal domain (FA58C), laminin G domains (LamG), calcium-binding EGF-like domain (EGF), fibrinogen-related domain (FReD), and putative band 4.1 homologues’ binding motif (4.1m). The CNTNAP1 amino acid substitution (XP_548083.3:p.Gly937Glu) position is shown together with the amino acid multiple alignment indicating the residue is highly conserved across species.

The across-breed GWAS using the discovery cohort of *ARHGEF10-* and *GJA9-* negative Leonbergers and Saint Bernards (144 cases vs. 373 controls) revealed a single genome-wide significantly associated region for LPPN (Figure 2a). The 15 best-associated markers were used to define a 4.6 Mb region of interest between 19.1 and 23.7 Mb on chromosome 9 (Supplementary Table S3). Fine-mapping of this region, using the available haplotypes from non-imputed SNP array genotyping data, included 87 markers centered on the best associated SNP (chr9:20,271,681). This revealed one homozygous haplotype present most frequently in LPPN-affected dogs (*n* = 21) of both breeds and not present in homozygosity in any of the controls (Supplementary Table S4). Therefore, the disease-associated region was narrowed to ~0.98 Mb (bp position 19,393,936 to 20,371,611), by a combination of sharing in the 21 homozygous cases from the discovery cohort (14 Leonbergers and 7 Saint Bernards), coupled with recombination events. Based on this analysis, we hypothesized that the causative variant explaining the GWAS hit was localized on this specific haplotype occurring in both breeds from the discovery cohort. Subsequent haplotype analysis of all 13,399 canids with available SNP genotype data provided evidence for the presence of this haplotype in total of 25 dog breeds (Supplementary Table S4).

### 3.4. Identification of the Candidate Causative Variant

In total, 38 protein coding genes and five lncRNAs were annotated within the disease-associated ~0.98 Mb critical interval on chromosome 9 (Figure 2b). Visual inspection of this region in the WGS of five LPPN-affected dogs homozygous for the associated haplotype (four Leonbergers and one Saint Bernard) revealed no evidence for the presence of structural variants. Filtering variants for homozygous alternative genotypes shared in these five dogs within the critical interval yielded 872 intronic or intergenic, 12 synonymous, and 18 protein-changing variants (Supplementary Table S5). In addition, WGS data were available for three Leonbergers out of the 55 dogs from the discovery cohort (Supplementary Table S1) carrying a single copy of the identified disease-associated haplotype (Supplementary Table S4). Filtering for heterozygous variants in complete linkage disequilibrium with this haplotype in three dogs reduced the number of putative variants to 93 intronic or intergenic, one synonymous, and one protein-changing variant (Supplementary Table S5).

*CNTNAP1* represents a functional candidate gene due to its involvement in human congenital hypomyelination, where vocal cord palsy is a common clinical finding [31], so we pursued the lone remaining missense variant in the *CNTNAP1* gene (CFA9:g.20298261C>T; c.2810G>A; p.Gly937Glu) further. This *CNTNAP1* variant was predicted to be deleterious by several prediction tools (MutPred2 score: 0.884, PROVEAN score: −7.667, PON-P2 probability for pathogenicity: 0.848). It is located in exon 18 of the *CNTNAP1* gene (Figure 2c) and affects a highly conserved amino acid residue at the end of the third laminin G domain of the CNTNAP1 protein (Figure 2d). Two missense variants (rs905697967:p.Gly938Arg and rs763033339:p.Gly938Glu) in the human *CNTNAP1* coding region at the corresponding position were found in the gnomAD [30]. Both variants were reported with allele frequency 7.95 × 10^−4^ and no homozygous individuals were detected [30]. The canine missense variant in *CNTNAP1* was present in 5 of the 688 control canid WGS with a frequency of 0.004 (Supplementary Table S5), including one homozygous (English bulldog) and four heterozygous dogs (golden retriever, Labrador retriever, English bulldog, and Kerry blue terrier).

### 3.5. The CNTNAP1 Variant Occurs in Several Breeds

Available SNP array genotype data of 13,337 dogs and 62 wild canids (Supplementary Table S1) were inspected for the identified *CNTNAP1*-associated haplotype (Supplementary Table S4). Out of this group, targeted genotyping by PCR and Sanger sequencing was performed in 2469 canids and demonstrated perfect concordance between the *CNTNAP1*-associated haplotype and the *CNTNAP1*:c.2810G>A genotype (Supplementary Table S4). In addition, 2362 dogs without SNP array data were directly genotyped for the variant (Supplementary Table S1); this included 557 dogs from the validation cohort and 1805 from the population cohort.

In total, the variant was found in 25 different breeds and no wild canids. Homozygotes for the missense allele in breeds other than the Leonberger, Saint Bernard, and Labrador retriever were identified in 46 English bulldogs, six Irish terriers, two boxers, one bullmastiff, one Peruvian hairless dog, one Yorkshire terrier, and one golden retriever (Supplementary Table S1), all with unknown precise health history. Additionally, two LPPN-affected mixed-breed dogs enrolled in the Michigan State University’s GOLPP study [6] were also homozygous for the missense allele (Supplementary Table S1).

Analysis of the validation cohort that included the three breeds with available health information (Leonberger, Saint Bernard, and Labrador retriever) demonstrated that the *CNTNAP1* variant is not present in a homozygous state in any dog apparently non-affected with LPPN (Table 2). The 18 homozygous LPPN-affected Leonbergers represent 4.1% of all as yet unexplained cases with any age of onset that were not carrying the previously identified disease-causing variants in *ARHGEF10* and *GJA9*. For the Saint Bernard, 10 out of 24 (41.6%) diagnosed dogs were homozygous mutant, whereas only 4.7% of GOLPP-affected Labrador retrievers carried two copies of the *CNTNAP1* variant. The homozygous A/A *CNTNAP1* genotype also occurred rarely in single dogs of the population controls from each of the three studied breeds (Table 2). Altogether, the mutant allele frequency was estimated as 6.6% in the studied Leonbergers (*n* = 2738 dogs), 13.9% in Saint Bernards (*n* = 305 dogs), and 5.2% in Labrador retrievers (*n* = 1524 dogs). Among the 22 other breeds segregating for this variant, the allele frequency was highest in the English bulldogs (*n* = 193 dogs) and Irish terriers (*n* = 184 dogs), estimated at 46.6% and 17.1%, respectively (Supplementary Table S1).

**Figure 3:**
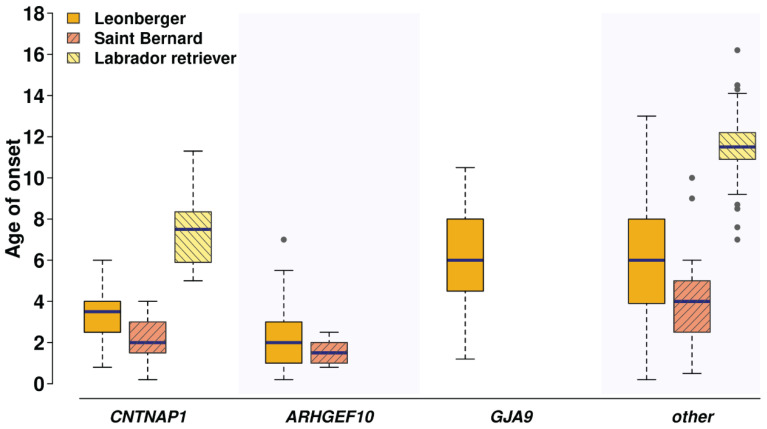
Age of onset of clinical signs for laryngeal paralysis and polyneuropathy (LPPN) differs depending on underlying genetic variants and across the three breeds. Comparison of the age of onset of clinical signs in the LPPN-affected dogs (*n* = 770), which were genotyped homozygous for the polyneuropathy-associated variants in *CNTNAP1*:c.2810G>A, or *ARHGEF10*:c.1955_1958+6delCACGGTGAGC [8], and homozygous or heterozygous for the variant in *GJA9*:c.1107_1108delAG [9], as well as the age of onset in the yet unexplained cases (other), is shown for Leonbergers (gold bars; *n* = 596), Saint Bernards (pink bars; *n* = 28), and Labrador retrievers (yellow bars; *n* = 146). Note that the *ARHGEF10* variant is only present in Leonbergers and Saint Bernards, and the *GJA9* variant only in Leonbergers.

Mean age of onset in the LPPN-affected dogs was investigated for a subset of 770 dogs with detailed health information from three breeds (596 Leonbergers, 28 Saint Bernards, and 146 Labrador retrievers) and showed a marked difference between the cases depending on the different underlying genetic causes (Figure 3). The average age of onset of clinical signs in the limited number of dogs homozygous for the herein described *CNTNAP1* variant was 3.4, 2.1, and 7.5 years in Leonbergers, Saint Bernards, and Labrador retrievers, respectively. In comparison, the age of onset of clinical signs in the previously characterized *ARHGEF10*-associated polyneuropathy [8] was seen in Leonbergers and Saint Bernards with average ages of 2.2 and 1.6 years, respectively. Additionally, affected Leonbergers with the *GJA9* frameshift variant [9] had average age of onset of their clinical signs of 6.2 years. Interestingly, the LPPN-affected Labrador retrievers that do not carry the herein identified *CNTNAP1* variant and come from the GOLPP study [6] showed a higher average age of onset of 11.5 years (Figure 3). The difference in age of disease onset between the dogs with the identified *CNTNAP1* variant and the cases without known disease-causing mutation was statistically significant in all three breeds (Leonberger *p*-value = 0.000001002, Saint Bernard *p*-value = 0.01681, Labrador retriever *p*-value = 0.002662). The difference between dogs with the *CNTNAP1* variant and the *ARHGEF10* variant was significant in Leonbergers (*p*-value = 0.002538) but not significant in Saint Bernards (*p*-value = 0.3095). The difference between Leonbergers with the *CNTNAP1* variant and the *GJA9* variant was statistically significant (*p*-value = 0.00000001797).

## 4. Discussion

This study has revealed strong evidence for a new potentially pathogenic variant associated with laryngeal paralysis and polyneuropathy (LPPN) initially observed in two closely related giant dog breeds, the Leonberger and Saint Bernard. Interestingly, the variant also explains some cases of GOLPP in Labrador retrievers and segregates at different frequencies in 22 other unrelated dog breeds, including English bulldogs and Irish terriers, suggesting that the derived allele predates modern breed formation. Apparently, the variant was not purged by either selection or drift.

The affected *contactin-associated protein 1* (*CNTNAP1*) gene has been previously implicated in human autosomal recessive neurological diseases with a broad spectrum of clinical phenotypes and neonatal and childhood onsets: congenital hypomyelinating neuropathy type 3 (OMIM 618186), lethal congenital contracture syndrome 7 (OMIM 616286), and childhood-onset Charcot–Marie–Tooth disease [32]. *CNTNAP1* is essential in the formation of paranodal axoglial junctions in myelinated axons and is also involved in regulating neural progenitor cells and the development of the cerebral cortex [33]. Pathological variants in the *CNTNAP1* gene may lead to defective or absent proteins critical to development of central or peripheral nervous systems. Even though, based on the current gnomAD [30] database, the corresponding glycine to glutamic acid exchange occurs very rarely in humans, so far, there is no evidence reported for disease association. Human patients with other *CNTNAP1* homozygous frameshift or nonsense variants show a more severe disorder with early-onset neurological disease, including severe respiratory compromise and early lethality, while those carrying missense variants can survive beyond infancy [34]. This suggests that the missense alleles affecting the myelination and development of paranodal junctions may be hypomorphic and have some residual function. Although the precise role of the protein domains in ligand binding is not fully understood, several missense variants were predicted to impact the domain structure and protein folding [34].

Pathological changes in semi-thin transverse resin sections of the peroneal nerves of three affected dogs had similarities to those in published human cases, including reduced myelinated nerves and inappropriately thin myelin sheaths for the axon diameters [35]; however, in the affected dogs, thinly myelinated fibers were scattered and fewer in number. This may reflect the severity of disease in neonatal onset human cases and milder disease with an adult onset in dogs. The increased population of small fibers in the affected Labrador retriever, as compared to the affected Leonberger, may reflect attempts at regeneration in the Labrador retriever or genetic differences in other modifying genes. There are several limitations to the pathological studies in these dogs: The number of affected dogs with available peripheral nerve biopsies was small; detailed study of the paranodal areas of the peroneal nerves was limited by the retrospective nature of the study, the use of archived nerve specimens obtained many years prior to the identification of the variant, and the necessity of preparation of the nerves at the time of original processing for teased fibers and for longitudinal evaluation; and the standard processing for diagnostic specimens in the laboratory of one of the authors (GDS) is in transverse section. Future in-depth prospective studies of the peripheral nerves, including laryngeal nerves in more cases of confirmed LPPN with the *CNTNAP1* gene variant in each breed, are necessary to fully evaluate the observed pathological changes.

The neurological diseases identified in humans associated with variants in *CNTNAP1* support our recent speculation, based on the enrichment of this allele in Leonbergers [7], that the herein-described missense variant predicted in silico to be deleterious represents a promising candidate causative mutation for inherited neurological disorders in dogs. The striking genetic association data implicate that this mutation affects the function of the encoded protein, although we have not studied this further. Homozygosity for the missense variant in the *CNTNAP1* gene is significantly associated with the development of LPPN in large and giant-sized dogs, indicating recessive inheritance in all three studied breeds (Leonberger, Saint Bernard, and Labrador retriever). As yet we do not have convincing evidence for causality in smaller dog breeds segregating for this variant, such as the English bulldog or Irish terrier, and further study with reliably phenotyped populations is needed. However, we hypothesize that the apparently higher allele frequency in English bulldogs may be a result of underdiagnosed LP due to breathing difficulties related to brachycephalic airway syndrome, including laryngeal collapse [36] obscuring a neurodegenerative LP. The observed higher allele frequency in Irish terriers, although without available health information, suggests that the association between the variant and LPPN phenotype is breed-specific and may not be pathogenic in some breeds although this needs to be evaluated. We also hypothesize that the observed later age of onset in the Labrador retriever group, compared to the Leonbergers and Saint Bernards, might be either due to the different genetic breed background and/or their smaller stature and correspondingly shorter laryngeal nerve length; the latter was previously suggested by correlation between growth (specifically height) and laryngeal neuropathy in horses [37].

## 5. Conclusions

In conclusion, we identified a potentially causative genetic variant in *CNTNAP1* associated with autosomal recessive younger-onset LPPN in large and giant dogs, specifically Leonbergers, Saint Bernards, and Labrador retrievers. Our results represent the first large animal model for a *CNTNAP1*-related neurodegenerative disease. The developed genetic test enables veterinary diagnostics and selective breeding against this deleterious variant across breeds to reduce the occurrence of LPPN. Therefore, selecting based on this additional disease-associated variant, which we have designated LPPN3, will enable dog breeders to make even greater strides in controlling the propagation of this devastating disorder and maintaining the health of Leonberger, Saint Bernard, and Labrador retriever populations. However, the fact that not all LPPN cases from the three intensively studied breeds carried the described variant, together with the broad range in age of onset of the clinical signs for the as yet unexplained cases, indicates that still unknown genetic heterogeneity of different forms of canine LPPN need to be studied in future.

## Figures and Tables

**Table 1:** Number of canids in each studied cohort.

Cohort ^1^	Dog Breed/Species	Total	Phenotype		
			LPPN-Affected ^2^	LPPN Non-Affected ^3^	Unknown
Discovery (*n* = 517)	Leonberger	426	126	300	0
	Saint Bernard	91	18	14	59
Validation (*n* = 1070)	Leonberger	859	500	359	0
	Saint Bernard	11	11	0	0
	Labrador retriever ^4^	200	150	50	0
Population (*n* = 14,174)	243 dog breeds	13,798	0	0	13,798
	Unknown/mixed heritage	314	0	0	314
	Wolf	58	0	0	58
	Golden jackal	2	0	0	2
	Andean fox	1	0	0	1
	Dhole	1	0	0	1

^1^ Additional details including the source of all data are available in Supplementary Materials Table S1. ^2^ Includes affected dogs with previously described laryngeal paralysis and polyneuropathy (LPPN)-associated variants in *ARHGEF10* [8] and *GJA9* [9]. ^3^ Includes dogs homozygous for the previously described leukoencephalomyelopathy-associated variant in *NAPEPLD* [14]. ^4^ Includes seven mixed-breed dogs enrolled in the Michigan State University’s GOLPP study [6].

**Table 2:** Segregation of the *CNTNAP1*:c.2810G>A genotypes with laryngeal paralysis and polyneuropathy (LPPN) in three breeds with available health information.

		*CNTNAP1* Genotypes		
Breed	LPPN Status	G/G	G/A	A/A ^1^
Leonberger (*n* = 2738)	Affected (*n* = 434) ^2^	358	58	18
	LPN1/LPN2 (*n* = 192) ^3^	180	11	1
	Non-affected (*n* = 659) ^4^	605	54	0
	Population controls (*n* = 1453)	1258	192	3
Saint Bernard (*n* = 305)	Affected (*n* = 24) ^2^	9	5	10
	LPN1 (*n* = 5) ^3^	2	3	0
	Non-affected (*n* = 14)	9	5	0
	Population controls (*n* = 262)	213	46	3
Labrador retriever (*n* = 1524)	Affected (*n* = 148)	132	9	7
	Non-affected (*n* = 45)	42	3	0
	Population controls (*n* = 1331)	1200	128	3

^1^ Includes the three herein described histopathologically confirmed cases. ^2^ LPPN-affected dogs that tested negative for *ARHGEF10* [8] and *GJA9* [9] mutations. ^3^ LPPN-affected dogs homozygous for the previously described polyneuropathy-associated variant in *ARHGEF10* (LPN1) [8], and homozygous or heterozygous for the variant in *GJA9* (LPN2) [9]. ^4^ Includes cases homozygous for the previously described leukoencephalomyelopathy-associated variant in *NAPEPLD* [14].

## Supplementary Materials

The following are available online at https://www.mdpi.com/2073-4425/11/12/1426/s1. Figure S1: Histogram showing the axonal size-frequency distribution of myelinated nerve fibers in peroneal nerve sections from control Labrador retrievers (average of three archived adult control dog samples), an LPPN-affected Labrador retriever, and an LPPN-affected Leonberger. Both affected dogs were homozygous for the *CNTNAP1* variant. Axons are distributed into bins determined by axonal diameter based on perimeter. Table S1: Sample designations and detailed information of all dogs used in the study. Table S2: Sample designations and affection status of all whole-genome sequences used for filtering variants. Table S3: The 10,000 most significant markers sorted by *p*-value obtained from the across-breed genome-wide association study. Table S4: Haplotype diversity for the LPPN-associated genome region on chromosome 9. Table S5: Variants in the region of interest obtained from GWAS on chromosome 9. Video S1: Video illustrating the clinical phenotype of two LPPN-affected dogs: a Leonberger and a Saint Bernard.
