# Supplementary figures and images for: A CNTNAP1 Missense Variant Is Associated with Canine Laryngeal Paralysis and Polyneuropathy
Source: Genes (Basel). 2020 Nov 27;11(12):1426. doi: 10.3390/genes11121426 (PMC7761076; doi:10.3390/genes11121426)

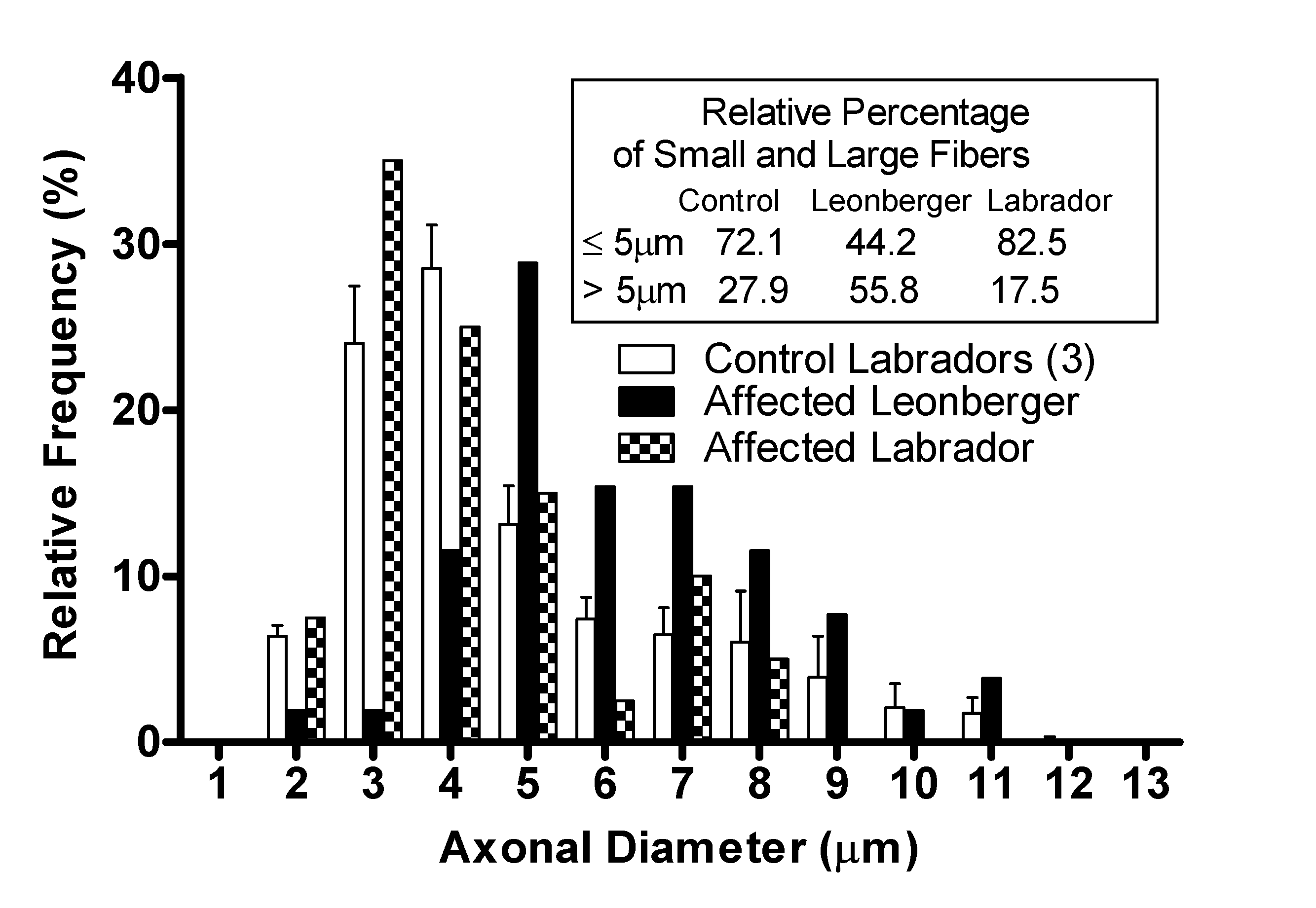

Supplement: Supplementary file 1 [file genes-11-01426-s001.zip › FigureS1_HistogramFigure.tiff]
